# Healthy dietary practices and its’ associated factors among adults of Nekemte dwellers, Oromia State, Western Ethiopia

**DOI:** 10.3389/fnut.2023.1259024

**Published:** 2024-01-24

**Authors:** Alemu Adeba, Dessalegn Tamiru, Tefera Belachew

**Affiliations:** ^1^Human Nutrition Department, Wallaga University, Nekemte, Ethiopia; ^2^Department of Nutrition and Dietetics, Faculty of Public Health, Jimma University, Jimma, Ethiopia

**Keywords:** health diet practice, prevalence, associated factors, adulthood, Nekemte

## Abstract

**Background and purposes:**

Appropriate healthy dietary practices are essential for well-being. Adopting of healthy lifestyle remains challenging worldwide. Ethiopia has an unacceptably high burden of malnutrition like other least developed countries. However, healthy dietary practices and their associated factors were not conducted in Nekemte town. Hence, the study was designed to assess healthy dietary practices and associated factors among middle-aged adults in Nekemte town from January 15 to February 30, 2019.

**Methods:**

A community-based cross-sectional study design was applied in Nekemte town. Primary data were gathered using a questionnaire from 266 adults and checked for normality. In both bivariate and multivariate logistic regression analyses the association and significance were determined at *P* < 0.05.

**Results:**

The Magnitude of dieting practice was 73.31% (unhealthy) and 26.69% (healthy), respectively. Being low-income households (*P* = 0.001), not married (*p* = 0.001), had a daily meal frequency [AOR: 1.91, 95% CI: (1.04, 2.71), and had poor knowledge of healthy diet AOR: 3.87, 95% CI: (3.23, 5.65)] were associated with unhealthy diets.

**Conclusion:**

The researchers identified unhealthy diet practices were widespread in the study samples of Nekemte populations. Hereafter, community-based lifestyle and Nutrition education through intensive participation of community leaders is highly recommended.

## 1 Introduction

A healthy diet can be defined as a pattern of food intake that has beneficial effects on health or at least no harmful effects. Unhealthy diets connote countries with scarce resources and from the four major risk factors of non-communicable diseases, unhealthy diet has been strongly associated with these diseases (1, 2). Many studies have shown that unhealthy diet not only increases the risk of metabolic syndrome, but also the potential risk factor for diseases like osteoporosis (3).

People follow dietary practices for different reasons at different times, such as: malnutrition prevention globally, usual dieting, holidays, celebrations, and out-catering, and they are defined as amalgamations of foods (4). This food may be a healthy diet or an unhealthy diet that has a positive or negative impact on the human body. Poor diet practices are habitual in low-income countries, and of the four major risk factors for non-communicable diseases, an unhealthy diet has been related to those illnesses (1, 2). Different researchers argued that this scenario is not only risky for metabolic syndrome development but also a potential risk factor for diseases like osteoporosis (3).

Whatever the recommended healthy diet intake is there, it is still under discussion in several cases. Only 42.56% of the countries in the world have their own dietary guidelines in world (5). Likewise, Ethiopia is at the initial stage of formulating the policy.

Evidence-based studies show that globally, people use the dietary diversity score to measure and indicate a good proxy of dietary quality (6–8). it is known that people in low- and middle-income countries typically eat fewer food groups than their staple foods, resulting in a low dietary diversity score (6). This is an arithmetical indicator of poor diet quality (6, 9).

Regarding food items, about 75% of the Ethiopian diet is cereal-based monotonous feed (7, 10, 11). Other studies reveal that the prevalence of low and medium dietary diversity scores among Ethiopian populations was 60% and 40%, respectively (12, 13).

Non-communicable Diseases (NCDs) are rapidly increasing globally and emerging radically in East Africa among adults (14, 15). Diet adequacies were formulated for different age groups of adults, but findings identified that many adults still do not follow a healthy diet. It is noted that there has been no such investigation regarding the topic based on adults in west Ethiopia of a specific age. Thus, the researchers are interested in investigating the prevalence of healthy diet practices and associated factors among middle-aged adults in Nekemte Town from January 15 to February 30, 2019.

## 2 Methodology

### 2.1 Narration of the study area

This study was conducted in the Oromia Region, western Ethiopia, at the hub of western Ethiopian Towns (Nekemte Town) to predict study populations’ healthy diet practices and their associated factors. The study is located 328 kilometers west of Addis Ababa.

### 2.2 Study design and period

A descriptive epidemiological study design, typically a community-based cross-sectional study was conducted to determine the status of dietary practices and their predictors among adults from January 15, 2019, to February 30, 2019.

### 2.3 Participants

All middle-aged (41–64 years) adults in Nekemte Town were selected as samples and adults unfit for selection criteria were not eligible currently for the research.

### 2.4 Sample size determinations

The sample size was determined by using the formula [n = [(Za/2)2*P (1−P)]/d2]. By considering the following assumptions: Za/2 = 1.96 at 95% confidence interval, a margin of error of 5%, and the most common prevalent is the component of metabolic syndrome among apparently healthy Ethiopian adults (with a proportion of 19.6% of central obesity) (16) which is; the final sample size was 266.

### 2.5 Sampling techniques

A probability sampling design was implemented for study participants. From six communities (administratively small sub-cities; locally termed Ganda or Kebele), one community was randomly selected by Systematic random sampling technique and the other one purposively. To ensure the relevance of the data, a third of the kebele must be selected. Additionally, another community was assigned that is not adjacent to the former Ganda or Kebele but has a similar socio-economic status. A simple random sampling method was applied to select study participants.

### 2.6 Data collection instruments

The data collection tool used well-structured Food Frequency Questionnaires. The FANTA and FAO (17, 18) a 7-day food-frequency questionnaire was used to assess dietary diversity score with twelve food groups. Questions contain socio-demographic and health diet mocks. The questionnaire can be implemented at the household or individual level, according to the purpose of the study. The HDDS indicator provides a glimpse of a household’s ability to access food as well as its socioeconomic status. Guiding Framework, Retrieved October 21, 2017, Method of Construction, the following 12 food groups are used to calculate the HDDS indicator: A. Cereals B. Root and tubers C. Vegetables D. Fruits E. Meat, poultry, offal F. Eggs G. Fish and seafood H. Pulses/legumes/nuts I. Milk and milk products J. Oil/fats K. Sugar/honey L. Miscellaneous; Each food group is assigned a score of 1 (if consumed) or 0 (if not consumed). The household score will range from 0–12 and is equal to the total number of food groups consumed by the household: Sum (A + to + L). The average household dietary diversity score for the population of study can be calculated as follows: Sum (HDDS)/Total number of households surveyed (5, 17, 18). Trained data collectors (five BSc Nurses and two MSc/MPH in Nutrition), including researchers, collected the information sequentially.

### 2.7 Reliability and validity test

The respondents were requested for their time prior to the main study or beta test. According to Mugenda and Mugenda (19), the reliability pre-test sample size can be between 1% and 10% of the total sample. Thus, 5% of the total sample was used as a pilot study to ensure reliability. A pilot test was carried out to evaluate the completeness, precision, accuracy, and clarity of the questionnaires; this ensured the reliability of the data collection instruments used (19). After the amendment of the final questionnaire, the researcher explained the purpose of the research and sought permission from the institution to carry out the actual research. The final questionnaires were distributed to the respondents with the help of research assistants. This enhanced the speed of data collection. Each completed questionnaire was treated as a unique case and a sequential number was given to each. Filling out the questionnaire took approximately 10 min. Prior to running the regression model, the existence of homoscedasticity, multi-collinearity, and normality assumptions was checked. A multivariate logistic model was used to isolate independent predictors of healthy dietary practices. The collected data was edited and entered into the Statistical Package for the Social Sciences (SPSS version 24) software to enable the carrying out of the analysis.

### 2.8 Data processing and analysis

The developed instrument for dietary practices was used to assess it (20). To assess dietary intake, 24-h multi-step recall was performed with FAO methodology, at least one food group in 7 days of a week. The collected data points were cascaded based on standards. The data were first checked for completeness and consistency, and cleaned for outliers and missing values. Adults’ mean dietary score from information gathered from respondents. Prior to running the regression model, the existence of homoscedasticity, multi-collinearity, and normality assumptions was checked. The researcher tested the model using the variance inflation factor (VIF). The predictors should be free of multicollinearity problems, most studies argue that if the mean VIF is less than 10, the model has no problem with multicollinearity.

The data was described using IBM software, (SPSS version 24). The findings were presented with frequency, percentage, and descriptive summaries used to explain the number of study participants in the analysis. Multivariable logistic regression analysis was performed to calculate the association between risk factors and significance level at a *P*-value of 0.05.

### 2.9 Ethical review and consent form

To conduct the study, ethical consideration was approved and taken from the Food and Nutrition Research Institute at Jimma University, Institutional Review Board (IRB) of the Institute of Health (Reference Number: IHRPGY/596/2019).

Prior to starting the study, an informed consent form was taken from the study participants. For illiterate respondents, the questionnaires were translated into their native language to understand the purpose.

## 3 Results

### 3.1 Subject characteristics

Of the 266 samples completed for the gender distribution, the majority of the respondents (186, or 69.93%) had unhealthy diet practices, and 62.78% were female. Among male participants, 71 (71.72%) had unhealthy diet practices. Findings revealed that 146 (54.89%) of the participants had a low income of 1.25 USD/44.45 ETB, and 209 (78.57) adopted unhealthy dietary habits. Similar to nearly three-fourths of participants, 187 (70.30%) of the adults were illiterate, and of the total illiterate participants, 134 (71.66%) had an unhealthy diet (Table 1).

**TABLE 1:** Socio-demographic of participants with distribution dietary practices, Nekemte (*n* = 266).

Variable	Categories	Dietary practices, n (%)	
		**Unhealthy**	**Healthy**
Sex	Female	115(68.86)	52(31.14)
	Male	71(71.72)	28(28.28)
	Total	186(69.93)	80(30.07)
Age group in years	41–48	119(82.10)	26(17.90)
	49–56	52(67.53)	25(32.47)
	57–64	29(65.90)	15(34.10)
	Total	200(75.19)	66(24.81)
**Income**			
(USD/person/day)	<1.25 USD	110(75.34)	36(24.66)
	>1.25 USD	99(82.50)	21(17.50)
	Total	209(78.57)	57(21.43)
Marital status	Not married	69(78.41)	19(21.59)
	Married	127(71.35)	51(28.65)
	Total	196(73.68)	70(26.32)
Educational status	Literate	51(64.56)	28(35.44)
	Illiterate	134(71.66)	53(28.34)
	Total	185(69.55)	81(30.45)

#### 3.1.1 The prevalence of healthy dietary practices

**FIGURE 1:**
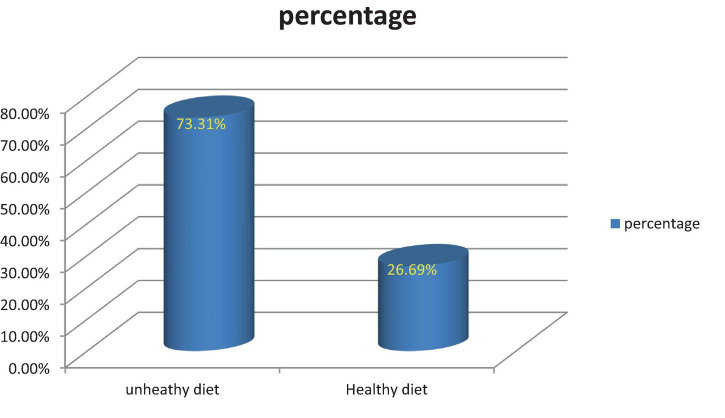
The prevalence of healthy diet consumed by respondents in Nekemte town (*n* = 266), 2019.

The overall prevalence of dietary practices assessed using dietary diversity score, for sure, indicated that 195 (73.31%) and 71 (26.69%) of respondents adopted unhealthy and healthy diets, respectively (Figure 1).

### 3.2 Factors associated with dietary foods

On bivariate logistic regression analysis: marital status, education, meal frequency, and income of participants demonstrate a relationship with the adoption of healthy foods among middle-aged Nekemte populations. Adults having low-income households, illiteracy, and meal frequency less than 3 times per day were significantly associated with unhealthy diet practices [AOR: 1.59, 95% CI: (1.37, 3.21), AOR: 3.20, 95% CI: (2.04, 5.98), AOR: 1.91, 95% CI: (1.04, 2.71), respectively]. The odds of having an unhealthy diet were almost three times (AOR = 3.87, 95% CI: 3.23, 5.65) higher for adults with poor knowledge of healthy diet compared to participants who did have that knowledge (Table 2).

**TABLE 2:** A multivariate logistic analysis findings of factors associated with a healthy diet (*n* = 266), 2019.

		Diet practice				
**Variables**	**Categories**	**Unhealthy**	**Healthy**	**COR**	**AOR(95% CI)**	***P*-Value**
Sex	Female	115	52	0.87(0.25,0.97)	0.54(0.48, 0.87)	0.868
	Male	71	28	1	1	1
**Income**						
USD/person/day	<1.25	110	36	0.65(0.39,0.86)	1.59(1.37, 3.21)	0.001**
	>1.25	99	21	1	1	1
Education	Illiterate	134	53	1.39(1.05,2.97)	1.20(1.04,5.98)	0.047*
	Literate	51	28	1	1	1
Marital status	Unmarried	69	19	1.46(1.13,4.85)	0.59(0.52, 0.98)	0.001**
	Married	127	51	1	1	1
Urban farming	Yes	5	17	0.07(0.05,0.76)	0.40(0.32, 0.58)	0.576
	No	203	45	1	1	1
Meal frequency	<3/day	148	35	22.19(19.25,26.97)	1.91(1.04,2.71)	0.000*
	≥3/day	13	68	1	1	1
Knowledge on a healthy diet	Poor	123	44	4.68(1.89,7.57)	3.87(3.23,5.65)	0.001**
	Good	37	62	1	1	1

Significances considered at *p < 0.05,

**p ≤ 0.001.

## 4 Discussions

This community-based study found that 73.31% of middle-aged adults adopted an unhealthy diet, which indicates a high prevalence. Similarly, 183 (68.80%) of the adults had three or fewer meal frequency consumption patterns per day.

According to Darmon and Drewnowski (21), the findings postulated that individuals with lower socio-economic status adapt and adopt unhealthy diets when compared to those with a higher one among adults in Australia (21). Likewise, the current study revealed that the healthy diet of participants was significantly associated (*p* 0.001) with the daily income of adults. This research outcome was also confirmed by studies done on Dietary intakes among US adults (22) and in Australia (23). Also, a study in the UK agreed that participants from households reporting lower financial or food security (since the start of the COVID-19 pandemic in the UK in February 2020) had poorer diets in some respects than participants from other households (24).

Having poor knowledge of healthy diets was strongly associated with having an unhealthy diet [AOR: 3.87, 95% CI: (3.23, 5.65)]. Similar to this finding, having good perceptions and valuable knowledge regarding the healthy diet concept is critically necessary for allowing people to make the “right life” choices. Another systematic review indeed suggests that nutrition knowledge is one of the factors that are most consistently related to a healthy diet (25).

Healthy food access is significant for improving population health (26). However; we found that many populations adopt unhealthy diets, which are highly prevalent at the study site. Independent variables showed a significant relationship with dependent ones among adults. Finally, this research shows that, in addition to confounding, the distortion of the association between diet and risk factors cannot be generalized unless entire populations adapt and adopt healthy diets.

This study has many strengths, but it also comes with different limitations. The study was limited by a smaller sample size, biophysical and biomarker characteristics of respondents were not considered. Besides that, cross-sectional studies have limitations. This study plays a crucial role in policy reviews, putting direction for implementers’ work on awareness creation for the adoption of a healthy diet and food security issues that boldly need great attention to measure the food quantity consumed with frequency. Besides, this study revealed that adult diet throughout life is masked, so future research perspectives will study inculcating rural and urban populations in a nationwide context using the evidence.

## 5 Conclusion

This research revealed that the prevalence of unhealthy diet practices was high (73.31%). And, majority of the participants had < 3 times the average meal frequency in a single day, and predicting variables were also associated with a healthy diet. The WHO recommends that people should eat a combination of different foods, including staple foods, legumes, vegetables, fruit, and animal source foods. On the contrary, almost all adults living in Nekemte Town practice cereal-based monotonous food. In recommendations, awareness creation about the adoption of a healthy diet, and food security issues we boldly need to pay attention to measuring the food quantity consumed with frequency. Besides, future research needs to study inculcating rural and urban populations nationwide.

## Data availability statement

The original contributions presented in the study are included in the article/supplementary material, further inquiries can be directed to the corresponding author.

## Ethics statement

To conduct the study, ethical consideration was approved and taken from the Food and Nutrition Research Institute at Jimma University, Institutional Review Board (IRB) of the Institute of Health (Reference Number: IHRPGY/596/2019). Prior to starting the study, an informed consent form was taken from the study participants. For illiterate respondents, the questionnaires were translated into their native language to understand the purpose.

## Author contributions

AA: Conceptualization, Data curation, Formal analysis, Funding acquisition, Investigation, Methodology, Project administration, Resources, Supervision, Validation, Visualization, Writing−original draft, Writing−review and editing. DT: Conceptualization, Data curation, Formal analysis, Funding acquisition, Investigation, Methodology, Project administration, Resources, Software, Supervision, Validation, Visualization, Writing−original draft, Writing−review and editing. TB: Conceptualization, Data curation, Formal analysis, Funding acquisition, Investigation, Methodology, Project administration, Resources, Software, Supervision, Validation, Visualization, Writing−original draft, Writing−review and editing.
